# Remote hearings in family courts in England and Wales during Covid‐19: Insights and lessons

**DOI:** 10.1111/fcre.12638

**Published:** 2022-03-11

**Authors:** Lisa Harker, Mary Ryan

**Affiliations:** ^1^ Nuffield Family Justice Observatory London UK

**Keywords:** COVID‐19 pandemic, family courts, remote hearings

## Abstract

The introduction of social distancing measures during the COVID‐19 pandemic resulted in family court hearings in England and Wales being conducted remotely, by video or telephone. Over a 15 months period, the Nuffield Family Justice Observatory undertook three rapid consultations to identify how remote proceedings were working, according to families and to a wide range of professionals who work in and around the family court. The consultations revealed the value of working remotely in certain circumstances, but also highlighted significant questions of fairness and justice. These insights should inform how courts operate in the future.

## Key points for the family court community

- Prior to the pandemic, the debate about conducting family law hearings by video or telephone were polarized into whether they were “good” or “bad.” There is now a more nuanced understanding of when it is and is not appropriate for hearings to take place over the phone or via video.
- While there are some definite advantages to remote hearings, there are also serious concerns about the fairness and justice of some hearings being conducted remotely.
- Many of the difficulties that parents and relatives had with being able to fully participate in remote court hearings are also likely to be evident in face‐to‐face hearings.
- There is value in “holding up a mirror” to be able to understand practice in family justice, bringing together a wide range of perspectives, particularly at a time of enormous change and challenge.

## INTRODUCTION

In March 2020, following the outbreak of the COVID‐19 pandemic and the introduction of social distancing measures, face‐to‐face hearings in the family courts in England and Wales came to an abrupt halt and were quickly replaced by telephone and video hearings.

The family justice system of England and Wales adapted quickly to working remotely. Given that there had long been plans to use information technology to improve the efficient running of hearings, the introduction of remote hearings heralded what some saw as a “new dawn” in the workings of the family justice system.

However, several weeks after social distancing restrictions were introduced, questions started to be raised about the fairness of operating hearings in this way (Kitzinger, 2020).^1^ Previous research also indicated that video and telephone hearings may be particularly difficult for certain groups of parents to access fairly (Byrom, 2020).^2^

In light of these concerns, the President of the Family Division (the head of the family justice system in England and Wales) asked the Nuffield Family Justice Observatory^3^ (an independent organization dedicated to improving the family justice system through research and insight) to undertake a rapid consultation in April 2020 to explore the impact of remote hearings.

At the time of the consultation in April 2020 it was not clear whether social distancing measures would be in place for very long, and so much of the discussion centered around which cases should be adjourned. However, by June 2020 it became clear that remote hearings were likely to remain in place for some time. The President noted in his message to the judiciary in June 2020^4^:

*Apparent potential unfairness which justified a case being adjourned for what was hoped to be a relatively short period of time, must now be re‐evaluated against this much longer timescale. The need to achieve finality in decision‐making for children and families, the detrimental effect of delay and the overall impact on the wider system of an ever‐growing backlog must form important elements in judicial decision making alongside the need for fairness to all parties. More positively, experience of remote hearings in the past two months has identified steps that can be taken to reduce the potential for unfairness……enabling cases to proceed fairly when previously they may have been adjourned* (para 6).

In light of this changing situation, the Nuffield Family Justice Observatory undertook a further rapid consultation in September 2020 to find out how remote hearings were operating. A third consultation was undertaken in June 2021, when social distancing measures started to be eased, to seek feedback as to which forms of remote working should endure beyond the pandemic.

This paper draws together the insights from these three consultations, which took place during an intense period of change for the family justice system in England and Wales.

## A NOTE ABOUT THE FAMILY JUSTICE SYSTEM IN ENGLAND AND WALES

Public law proceedings are proceedings brought by the state because a child has suffered or is at risk of suffering significant harm. Proceedings are brought by local authorities who have responsibility for child protection. In these proceedings parents and children are entitled to legal representation and the court is also assisted by children's guardians from the Children and Family Court Advisory Service (Cafcass). Private law proceedings concern family disputes between family members, usually parents. People are not entitled to legal aid to cover representation in such proceedings, except where one party is seeking protection as a result of domestic abuse. As a result, many people in private law proceedings are unrepresented. They are referred to as litigants in person. Family proceedings can be heard by judges of different levels of seniority and also by lay magistrates who are assisted by special legal advisers.

## METHOD

### Consultation design

Each consultation was conducted over a two to three‐week period. The first consultation was undertaken from April 14–28, 2020, the second from September 10–30, 2020, and the third from June 10–27, 2021.

In the first consultation, survey participants were asked:

- Have you had direct experience of a remote hearing?
- If yes, what sort of hearing was it, which court center was involved, through which remote method was it conducted and what was your role?
- What factors worked well?
- Did you have any concerns?
- If you have concerns, do you consider that this way of working was justifiable in the short term?
- How could the experience be improved in dealing with the current crisis?
- Have you had any direct feedback from lay clients or third parties (intermediaries/interpreters/experts) as to their experience of the remote hearing?
- Are you happy to be contacted for further questions?

Participants were invited to either submit written responses to a dedicated email address, or to provide their responses over the telephone. Groups supporting parents in legal proceedings were contacted directly to ask them to encourage parents to respond.

In the second and third consultations, respondents were invited to complete an online survey. Two separate surveys were designed, one for professionals and one for parents/relatives. In both surveys, respondents were asked about their experience of remote hearings, the technology used, their views about whether they thought the remote hearing had been fair, what had or had not worked well, and their recommendations for improvements. Some basic demographic information was also collected. The survey for parents was shorter and less technical compared to the one for professionals, focusing on how they had taken part in their hearings, whether they had understood the process and whether they had thought it was fair. The third consultation was designed to inform activity and planning as the family justice system began its own “recovery” from the pandemic, so professionals were asked for their views about whether specific types of hearings could continue to be held remotely.

The questions for each survey were drawn up by the small team responsible for analyzing the surveys and writing the reports. In designing the surveys, the teams were informed by the issues emerging via national and social media reporting, published information for courts, and legal professionals and through informal discussions with family justice professionals. Respondents were encouraged to provide qualitative as well as yes/no responses.

### Participants

All three consultations sought feedback from parents, other family members, and all professionals in the family justice system including judges, magistrates, barristers, solicitors, Children and Family Court Advisory and Support Service (Cafcass) advisers, court staff, and social workers.

Those who had experience of a remote hearing in public or private law family proceedings were invited to respond. This included experience of any type of remote hearings (including administrative hearings, interim hearings, and final hearings). Early hearings at the start of proceedings where a timetable is set and directions made about the filing of evidence, are often referred to as administrative hearings. Interim hearings take place during proceedings and involve the making of interim orders, for example in relation to contact with the child or in relation to where the child should live. These hearings can be contested and involve evidence being given. Final hearings take place at the end of the proceedings.

Survey respondents were recruited through extensive online publicity, including the Nuffield FJO website and social media, supported by the office of the President of the Family Division and relevant professional organizations. In addition, several events were held to provide an opportunity for people to share their views and experiences. Organizations (such as the Association of Lawyers for Children and The Association of Her Majesty's District Judges) also conducted their own consultations and were willing to share the information that they had gathered.

To ensure that the views of parents were sufficiently represented, particular efforts were made to encourage feedback from parents and litigants in person by contacting organizations that support parties through the legal process. In addition, a focus group with parents and kinship careers was held as part of the first consultation. As part of the second consultation, Nuffield FJO commissioned the Parent Families and Allies Network (PFAN) to collect information from parents and relatives through focus groups and interviews. Three focus groups and 10 interviews were held with a total of 21 parents. PFAN provided notes of the interviews and focus groups, as well as some background information about the parents who participated, including the type of proceedings they were involved in and where (geographic region) their case was heard. For the third consultation, parents who had responded to previous surveys and had indicated a willingness to be contacted again were sent a personal message and asked to respond once more if they had been involved in further hearings since September 2020 and organizations supporting parents were once again asked to encourage parents to respond.

Nine hundred and thirty‐two participants (professionals and parents) responded to the first consultation, 1306 responded to the second consultation and 3219 responded to the third consultation. Some responses collated information from groups of individuals, but this was counted as a single response, so the actual number of individuals who provided feedback was higher than recorded.

There was a similar pattern in the type of respondents to the consultations, with judges, barristers, solicitors and magistrates making up at least two‐thirds of the respondents in each consultation (see Table 1 below).

**TABLE 1:** Type of respondents

	Consultation 1	Consultation 2	Consultation 3
	*n* = 932	*n* = 1306	*n* = 3219
Judges	15%	15%	10%
Magistrates	8%	17%	13%
Legal advisers to magistrates	4%	5%	3%
Barristers	26%	15%	25%
Solicitors	15%	13%	27%
Social workers	5%	7%	4%
Cafcass staff	18%	10%	6%
Parents	3%	9%	5%
Other (e.g., working for Family Drug and Alcohol Courts, intermediaries, voluntary organizations)	6%	8%	6%

Responses were received from all regions of England and Wales and there was a reasonable geographical spread.

### Analysis

In the first consultation, responses were all qualitative. The research team was made up of people with long experience of the family justice system. Basic information in relation to role, type of hearing or hearings and technology used was entered onto an excel spreadsheet, along with whether the overall experience of remote hearings had been positive, negative or mixed. In the second and third consultations responses were received through the online Microsoft Forms platform in Excel files which enabled quick analysis of quantitative data at the aggregate level. Analysis of the qualitative responses in all three consultations was carried out by the research team. Emerging themes were identified from the first 100 responses and agreed across the team. Thereafter, quotes from respondents illustrating these themes were selected. There was constant communication between the team to ensure any new themes could be identified quickly. Appropriate procedures were used to ensure data remained confidential, so no individual respondent was identifiable.

### Study limitations

These were rapidly conducted surveys relying on those who chose to respond. Each survey included slightly different questions. They were analyzed at speed with a focus on identifying main messages and with little opportunity for deeper investigation of variables. Despite these limitations, given the large number of participants the consultations provided a valuable snapshot of the experience of remote hearings for professionals and families within the family justice system in rapidly changing circumstances.

## FINDINGS

### Type of proceedings

Respondents' experience of remote hearings reflected changing patterns during the pandemic. At first public law cases were prioritized, so it was unsurprising that 43% of respondents to the first consultation had experienced a remote hearing in relation to public law (only) and 20% had done so in relation to private law (only). 24% of respondents had experienced remote hearings in both public and private law (and in 12% of cases it was unclear what the experience was).

When it became clear that social distancing measures were likely to last more than a few months, all types of family law cases started to be held remotely. By the time of the second and third consultations more respondents (57% and 55% respectively) had experienced remote hearings in both public and private law. Similarly, by the time of the second and third consultations, many more respondents had experienced interim or final hearings where evidence had been given and cross examination had taken place, in contrast to the first survey where experience of final hearings was limited.

### Representation

In the second and third consultations, parents and relatives were asked if they ever had legal representation. 42% of parents who responded to the second consultation, and 46% of parents who responded to the third consultation, had attended hearings without legal representation.

Many of the professionals reported hearing or attending cases where one or more of the parties were unrepresented (usually both the mother and the father were unrepresented). For example, 48% of professionals who responded to the second consultation said that half or more cases that they had heard or attended involved unrepresented parties (litigants in person).

### Technology used

A wide range of different telephone and video platforms were being used to conduct remote hearings. Initially most hearings were conducted by telephone, although most respondents stated a preference for hearings to be conducted by video. Even 15 months into the pandemic, although access to video conferencing had increased, telephone hearings continued to be widely used for final and contested hearings as well as for administrative and direction hearings.

Responses indicated variations in the use of technology across courts in different geographic locations and variations depending on the level of judge presiding over the hearing and in some cases the personal preferences of the presiding judge or magistrates.

In the summer of 2020, the court service in England and Wales introduced a new video platform to all family courts (Cloud Video Platform). However, by September 2020, only just over half (55%) of family justice professionals had used the new platform. By June 2021 this had risen to 78%. However, respondents also reported continuing to use other types of video platforms, as well as variety of telephone platforms.

### Overall views of remote hearings

The respondents to the first consultation were evenly balanced in terms of their overall positive and negative reactions to remote hearings. This reflected the fact that many respondents felt that remote hearings were justified in some cases in the circumstances of the pandemic, and, for some types of hearings, were even preferable, but they had serious concerns in relation to other types of cases, particularly those where the issue was complex and/or contested and evidence was required, or those where final orders were being made, especially where vulnerable parties were involved. It was clear from the responses that there was a wide variation in practice and no consistency in decisions about which cases should be adjourned.

By September 2020, when remote hearings had been operating for 6 months, most professionals who responded to the second consultation felt that things were working more smoothly, either all of the time or some of the time. Professionals continued to report that there were also some benefits to working remotely, for professionals and parties, particularly in relation to a reduction in time spent traveling or in waiting at court for a case to be called on.

However, responses to the second consultation also revealed a clear disconnect between the experience of professionals and that of parents and other family members in relation to remote hearings. While many professionals were positive about remote hearings, the majority of parents and family members had concerns about the way their case had been dealt with, and just under half said they had not understood what had happened during the hearing. Many professionals, although positive about remote hearings for some types of hearing or case, remained very concerned about the experience of vulnerable lay parties and litigants in person.

By June 2021, we were able to elicit detailed feedback about the suitability of remote hearings being conducted in specific types of cases or circumstances. The majority of professional respondents saw a continuing role for certain types of remote hearing, although many felt that the decision should be made on a case‐by‐case basis. The main considerations respondents identified as relevant to such a decision were the vulnerability of lay parties and their wishes and views, the complexity of the case, and whether there was access to suitable technology for all those taking part.

Overall, there was support for remote “administrative” hearings (subject to certain caveats) such as case management hearings, first hearing dispute resolution appointments^5^ and also for initial and/or ex parte applications for non‐molestation/occupation orders.^6^ There was much less support for remote fact‐finding hearings, hearings involving contested applications for interim care or contact orders, or final hearings.

Many barristers, solicitors, local authority lawyers, social workers and Cafcass workers highlighted the positive impact of remote hearings on their time and working patterns.

There were parents who reported that they had preferred the remote hearing from having to attend court and others who were pleased that at least their hearing had taken place. There were mixed views from women who had experienced domestic abuse. For some, being able to avoid going to court and potentially meeting the perpetrator was a relief, but for others the experience of seeing or hearing the perpetrator in their home via phone or video was very upsetting. Women who had been able to make use of special measures when in court (giving evidence from behind a screen) were not always able to ensure that they would not be shown giving evidence while taking part in a video hearing.

In the main however, it was clear that parties to cases were far less positive about remote hearings than professionals. A clear majority of parents (83%) who responded to the third consultation had concerns about the way that their case had been dealt with. Many expressed concerns that were apparent before the onset of the pandemic and remote hearings, particularly in private law. These included: concerns that judges and lawyers do not have a good understanding of domestic abuse and, in particular, of coercive or controlling behavior; judges are misogynistic; courts do not properly understand parental alienation; courts are biased against fathers; dissatisfaction with Cafcass; and frustration at the expense of legal representation and the lack of availability of legal aid. Many other parents, relatives, and lay parties raised concerns specifically about their experience of remote hearings. The majority of these comments were about the fairness and the smooth working of remote hearings, or concerns about technology or delays in proceedings.

### Concerns about fairness

Significant concerns were raised by all respondents (not just parents) to all three consultations about the fairness of remote hearings in certain cases and circumstances. There were also some worrying descriptions of the way some cases had been conducted.

The concerns included the difficulties in reading reactions and communicating in a humane and sensitive way when there was no face‐to‐face contact. Many professional respondents referred to the importance of being able to show empathy when involved in family justice cases, particularly in public law cases where children may be removed from their parents.

The court process is more important than simply being an administrative adjudication. It's a very human set of interactions. My role as a judge is absolutely dependent on the humane administration of a very, very complex interactive process (Judge).

There were also concerns that parents were having to take part in remote hearings alone and from their homes, without any legal or other emotional support. It was noted that it was difficult to assess vulnerability before a hearing took place:

*We cannot always identify whether a person is going to be vulnerable. In the middle of hearing yesterday a mother told me she was self‐harming. I immediately adjourned the hearing and an ambulance was called. My concern was that I had not identified the hearing as unsuitable for remote and I did not pick up on the problem until the mother told me. We have a good triage system but it didn't pick this up* (Judge).

In addition, respondents noted that it could be very difficult for parents to ensure privacy and confidentiality when they were calling from home. In the earlier phase of social distancing, when schools were closed, it was not uncommon for parents to be at home with the children who were the subject of the proceedings:

*The mother, who was at risk of having her four children removed, gave evidence by telephone from her garden shed as there was nowhere else private she could go as she was self‐isolating due to COVID‐19 and the children were in the house, being cared for by their grandmother. It was unsatisfactory to make any decision without being able to assess the evidence in the round, and unsatisfactory for the mother to give such important evidence in these circumstances. The likelihood of parents involved in care proceedings having a private space from which to attend remote hearings seems low (Judge*).

The concerns about parents being alone and unsupported during remote hearings where important decisions were being made were particularly strong when the case involved an application to remove a newborn baby from the mother.

[It] was a contested removal hearing in respect of a newborn baby. The mother has a history of local authority involvement with two other children. I represented her. I found it profoundly inappropriate not to be speaking to her in person but on a telephone when she had never met me before and had given birth to her baby only the previous day. The court decided, almost inevitably, that the child should be removed but for that mother to be listening to the hearing in a side room in hospital, and to be told of the court's decision by telephone (she missed the judgment because she had been called away to feed the baby by a midwife who came in to the room where the mother was) without me being there to put an arm round her seemed horribly cruel to me (Barrister).

Another issue identified by both parents and professionals was the problem of communication between lay parties and their legal representatives before hearings and during hearings. Pre‐hearing communication, instead of being in person, was mainly by phone or email which adversely affected the taking of instructions and drafting of statements. During hearings it was difficult for many parents to communicate with their legal representatives in private because they did not have access to more than one device to enable them to send private messages, and because judges or magistrates did not always allow for breaks or adjournments so that lawyers could communicate with their clients. Particular concerns were commonly raised in relation to parties with a disability or cognitive impairment or where an intermediary or interpreter was required.

In public law care proceedings, it is common for an interim care order to be made, authorizing the removal of the child from the parent until the court reaches a final decision. Pre‐pandemic, such orders were accompanied by contact orders allowing the parents regular face to face contact with their child, several times a week. This was important in all cases but particularly important in cases involving babies. During the pandemic, while rules relating to social distancing were in place it was rare for parents to be able to see their children in person. Arrangements were made for digital contact, which was helpful with older children, but there were considerable concerns expressed by family justice professionals at the impact on the developing relationships between parents and babies where face to face contact could not take place and the subsequent impact on the outcome of proceedings.

*Contact has generally been virtual which in my view is inadequate. The children are not able to develop or maintain a bond with their parent on a screen ‐ particularly young babies and toddlers. It can be very confusing for the children* (Barrister).

There was widespread concern for litigants in person in private law matters. Many examples were given of support being provided to litigants in person by judges, magistrates, and legal advisers, but it was acknowledged that this was often insufficient.

*I have major concerns about the fairness of proceedings with both Litigants in Person and some represented parties (e.g., parents with learning type difficulties in care cases) in remote hearings. Removing children and making major decisions, e.g., on contact, can have long term (even lifelong) consequences. Notwithstanding everyone's best efforts remote parties cannot sometimes be adequately engaged and whilst Judges and professionals can ‘get on with it’ and make it work I have real misgivings about how fair it is and how fair it is felt and seen to be* (Judge).

While concerns were expressed most strongly by parents and relatives who have experienced remote hearings, it was clear that judges, lawyers and social workers had concerns too. Although 6 months into the pandemic most family justice professionals felt that fairness and justice had been achieved in the cases they were involved with most or all of the time, it was clear from responses that professionals also had concerns about whether proceedings were perceived as fair by parties in all cases. Professionals shared concerns about the difficulties of being sufficiently empathetic, supportive, and attuned to lay parties when conducting hearings remotely. By June 2021, 63% of professionals who responded to the consultation felt that further arrangements needed to be in place to make remote hearings fair and work smoothly.

*Let's not kid ourselves ‐ none of us would have thought these methods of working achieved fairness and justice six months ago* (Judge).

*I think they have been fair and just in terms of legal outcome but I am not sure the perception has always been of fairness and justice being done* (Judge).

### Impact on the Authority of the Court

Many respondents raised concerns about the impact that working remotely was having on the formality and authority of the court. Family justice professionals expressed concern that the relative informality of telephone and video hearings meant that lay parties were not taking the court as seriously as they would if the hearing were taking place in person. This was not an issue identified in the consultation undertaken in April 2020 but was voiced by respondents to the consultations undertaken in September 2020 and June 2021.

Professionals reported changes in lay party behavior due to hearings being held online.

*Lay parties often don't treat the court process with the usual respect when connecting from home. I have undertaken cases where a lay party is in bed, or in pyjamas or trying to do household tasks while participating* (Barrister).

Parents also expressed concern about professionals appearing overly relaxed in situations where important decisions are being made about their future.

*The social worker was zipping in and out … and that wouldn't have happened in a court they would have had to be sitting there in silence* (Mother, Parent Families and Allies Network).

There were concerns that the relative informality of proceedings meant that parents did not realize the seriousness of the decisions being made until it was too late.

*The respect and authority of the court is being slowly eroded. The quality of evidence mixed. The informality of the home setting undermining the seriousness of the process. Often the hearings seen as ‘call’ not a hearing – counsel being seen not in court attire, witnesses and parties having mugs of tea when they think they are not on view ‐ getting up and walking about when not speaking ‐ clearly attending to other matters – e.g. emails whilst in the hearing* (Judge).

### Problems with technology and administration of hearings

Many of the concerns relating to fairness and upholding the gravitas of the court were caused, or exacerbated, by problems of managing telephone and video technology, or difficulties with the administration of remote hearings.

The responses to the three consultations suggested that the technology to support remote hearings did improve over time. Nevertheless, many respondents continued to report difficulties. Respondents' concerns about video technology normally related to the quality of connection and access to appropriate hardware (screens and loudspeakers). These difficulties affected professionals as well as parents, but the majority of concerns related to the difficulties parents experienced in fully participating in hearings. The pandemic highlighted issues of digital poverty as it became clear that many parents in public law care proceedings did not have access to laptops, Wi‐Fi, or smart phones. Where there was no access to Wi‐Fi, they often had very limited access to data, making it difficult for them to take part in online hearings.

Many of my clients may not have Wi‐Fi, no credit on their phones, phones that are infrequently charged and no access to laptops nor iPads. They live in social deprivation, and their housing may be shared or not sufficiently private for a hearing to be conducted (Barrister).

The vast majority of parents received no help in accessing the technology to take part in the hearing. Professionals expressed concern about the lack of clarity around responsibility for supporting lay parties with access to technology and/or private space from which to join hearings remotely. As a result, it was clear that practice across England and Wales was very varied in terms of the support available.

It was also clear from responses to the consultations that the family court system in England and Wales lacked consistent high‐quality IT equipment and connectivity. Common problems arising from this identified by respondents included difficulty in hearing people, difficulty seeing people, and difficulty identifying who is speaking.

Concerns were also raised about the extent of professionals' technological capabilities, a lack of opportunities for training and limited IT support available to judges and magistrates.

The limited IT support available meant that judges, magistrates, and legal advisers were spending considerable amounts of time trying to deal with problems that arose, which impacted on delays in the system. It also resulted in technical issues during hearings which impacted on the ability of lay parties to follow and understand what was happening.

Both consultations revealed wide variations in practice across different courts and geographic areas in terms of how hearings are organized. This included the information sent to lay parties and professionals about how the hearing would work, the timing of sending out notifications of a hearing, the timing of the decision about what technology would be used for the hearing and arrangements for enabling professionals and lay parties to contact the court if there were any connectivity problems.

*I sit in one large court and one small court. The smaller court is very badly organised and has to be prompted in relation to each case to make arrangements. There is no awareness of what constitutes a fair remote hearing shown by the office staff. The larger court has systems in place which result in daily lists being presented with all necessary information, video hearings set up and supported on the basis of directions given and electronic bundles supplied. This is much more effective. I suspect that the difference is due to leadership* (Judge).

## RECOMMENDATIONS MADE

Each consultation asked respondents for recommendations about how to improve the fairness and smooth running of remote hearings. The responses were remarkably similar across all three consultations.

Many requested measures that would make hearings run better, such as guidance for lay parties about the court process and the format of the hearings, better support to access the relevant technology and clarity about who was responsible for providing assistance to lay parties and parents. Some of the recommendations for improved practice were remarkably modest, such as being sent the links of hearings the day before, having an usher to manage attendance, and ensuring all bundles of evidence have been sent out in advance. Some of the requests for improvements in the way hearings were run were relevant to any hearing, not just those run remotely: making sure everyone has joined the hearing, making sure to identify all the different people involved, checking everyone can hear and/or see, checking whether people need a break to communicate.

There were also many requests for improvements in technology, such as ways to assist with communication between lay parties and their legal representatives during a hearing and improved technology to assist with interpreters and intermediaries. Judges and magistrates also requested better technical support, as well as improvements to the technology itself (better hardware, better sound, better connectivity).

## DISCUSSION

It is often noted that family justice professionals have only a limited view of the family justice system. While they have sight of individual cases, they may not be aware of practice in other geographical areas, in other professions, or even within their own profession. The consultations demonstrated the value of “holding up a mirror” to be able to understand practice, particularly at a time of enormous change and challenge. It was especially valuable to be able to bring together a wide range of perspectives – including judges, lawyers, social workers, Cafcass workers, parents and other relatives.

The President of the Family Division of England and Wales commented on the power of this process to inform practice:

I am confident that all who are interested in Family Justice at this time will read [it]….The process of research has held a mirror up to what we are currently doing. I hope that its publication will stimulate informed discussion and debate. The process of reading the document, and seeing what is said there, may well act as a corrective for future hearings – either by identifying occasions when a remote hearing may have been less than satisfactory, or by flagging up suggestions for improvement – in a more subtle and effective manner than any formal guidance might achieve.^7^

The findings of both reports were subsequently presented to over 1000 judges and magistrates as part of the training programme routinely offered to them.^8^

Prior to the pandemic, the debate about conducting family law hearings by video or telephone were polarized into whether they were “good” or “bad.” These consultations contributed towards a more nuanced understanding of when it is appropriate for hearings to take place over the phone or via video and what problems need to be addressed to make sure such hearings work well.

As of October 2021, we are not able to judge whether the process of doing so has influenced practice across the family justice system, in the way that the President of the Family Division envisaged. Courts are beginning to reopen and more face‐to‐face hearings are starting to be heard, but it is too soon to judge whether the insights gleaned by the consultations have had a lasting impact on the way hearings are organized and run.

Nevertheless, it does suggest that the process of “holding up a mirror” to the family justice system in this way may be valuable in the long term, not only during a crisis. It was particularly striking that the consultations exposed the difficulties that parents and relatives had with being able to fully participate in court hearings, whether they were parties in public law proceedings or litigants in person. While the nature of remote hearings made participation especially difficult, many of the problems reported by parents and relatives are equally likely to be evident in face‐to‐face hearings: not feeling “heard,” not fully understanding the process etc. As one senior High Court judge commented:

This [the NFJO's research] has led me to reflect that perhaps this divergence between what lawyers experience and what litigants experience is not confined to the remote process. In fact, I am sure it is not. It has led me to think more about what litigants must feel going through the family justice system in normal times. It seems to me that … we need to think about how we make it a fairer and more humane process for litigants in the family justice system both for those who attend hearings remotely and those who come back to court. Quite a profound lesson, I think, for all of us.^9^

## Footnotes

^1^
Celia Kitzinger, *Remote Justice: a family perspective*. transparency project (Mar. 29, 2020), https://www.transparencyproject.org.uk/remote-justice-a-family-perspective/ (accessed Sept. 29, 2021).

^2^
Natalie Byrom, *What do we know about the impact of remote hearings on access to justice: a rapid evidence review*, nuffield family justice observatory. (May, 2020), https://www.nuffieldfjo.org.uk/resource/impact-remote-hearings-access-to-justice.

^3^

nuffield fam. just. observatory, www.nuffieldfjo.org.uk.

^4^
Sir Andrew McFarlane, *The Family Court and COVID‐19: The Road Ahead*, courts and tribunals judiciary (June 9, 2020), www.judiciary.uk/announcements/the-family-court-and-covid-19-the-road-ahead/ [Accessed Sept. 29, 2021].

^5^
These are first hearings in proceedings, (CMH public law, FHDRA private law) setting timetables and giving directions about the filing of evidence

^6^
Initial hearings in private law proceedings in cases of domestic abuse where the victim is seeking protection are frequently heard ex‐parte with an early return date.

^7^
Id.

^8^
James Munby, *Making remote hearings accessible, fair and humane under COVID 19*, nuffield fam. just. observatory (Feb. 18, 2021), https://www.nuffieldfjo.org.uk/events/webinar-remote-hearings-fair-covid-19.

^9^
Id.

